# Conformation specific antagonistic high affinity antibodies to the RON receptor kinase for imaging and therapy

**DOI:** 10.1038/s41598-022-26404-7

**Published:** 2022-12-29

**Authors:** Xin Yu Koh, Xiao Hui Koh, Diana Spiegelberg, Preeti Jha, Marika Nestor, Le-ann Hwang, Ban Xiong Tan, David Philip Lane

**Affiliations:** 1 Disease Intervention Technology Lab, Institute of Molecular Cell Biology (IMCB), Agency for Science, Technology and Research (A*STAR), 8A Biomedical Grove, #06-06, Neuros/Immunos, Singapore, 138648 Singapore; 2 Experimental Drug Development Center (EDDC), Agency for Science, Technology and Research (A*STAR), Singapore, Singapore; 3 Department of Immunology, Genetics and Pathology, Uppsala University, Uppsala, Sweden; 4 Department of Surgical Sciences, Uppsala University, Uppsala, Sweden; 5 Department of Radiology, University of Texas Southwestern Medical Centre, Dallas, USA

**Keywords:** Immunotherapy, Diagnostics, Cancer imaging, Cancer screening, Cancer therapy, Oncogenes

## Abstract

The RON receptor tyrosine kinase is an exceptionally interesting target in oncology and immunology. It is not only overexpressed in a wide variety of tumors but also has been shown to be expressed on myeloid cells associated with tumor infiltration, where it serves to dampen tumour immune responses and reduce the efficacy of anti-CTLA4 therapy. Potent and selective inhibitory antibodies to RON might therefore both inhibit tumor cell growth and stimulate immune rejection of tumors. We derived cloned and sequenced a new panel of exceptionally avid anti-RON antibodies with picomolar binding affinities that inhibit MSP-induced RON signaling and show remarkable potency in antibody dependent cellular cytotoxicity. Antibody specificity was validated by cloning the antibody genes and creating recombinant antibodies and by the use of RON knock out cell lines. When radiolabeled with 89-Zirconium, the new antibodies 3F8 and 10G1 allow effective immuno-positron emission tomography (immunoPET) imaging of RON-expressing tumors and recognize universally exposed RON epitopes at the cell surface. The 10G1 was further developed into a novel bispecific T cell engager with a 15 pM EC50 in cytotoxic T cell killing assays.

## Introduction

The RON receptor tyrosine kinase (MST1R) is expressed at high levels at the surface of many tumor cells of epithelial origin. Overexpressed RON is an oncogenic driver and small molecule inhibitors of the RON kinase and antibodies to the extracellular domain of RON have shown anti-tumor activity in a variety of pre-clinical models^1^. RON is a member of the MET receptor family and its sole ligand is the macrophage stimulating protein (MSP)^2^. RON is expressed on myeloid cells and recent studies have shown that the anti-tumor activity of anti-CTLA4 antibodies is enhanced in RON kinase domain knockout mice, suggesting that the inhibition of RON may enhance the activity of anti-CTLA4 treatment in enhancing host anti-tumor immunity^3^. Narnatumab, a humanized monoclonal against RON entered phase I clinical trials but failed for the lack of efficacy, in part because the antibody could not be given at high doses due to solubility issues^4^.

Several groups have reported the use of an antibody to target RON expressing tumours, but total tumour abolition have not been reported with the naked molecule alone. This may be due to a much lower level of RON receptor expression on cancer cells as compared to the other tyrosine kinases like HER2 and MET^5^. However, the advantage of targeting RON is its superior safety profile as compared to the other tyrosine kinases as it has much lower expression on normal tissues as well^6^. This allows for the adoption of a strategy to target RON with a more potent design, without the fear of inducing unmanageable “on target off tumour” toxicities. Using an antibody drug conjugate design, RON targeting ADCs like Zt/g4-MMAE achieved complete tumour abolition responses with limited toxicities^7,8^. However, antibody drug conjugates historically have their own set of challenges including unstable linkers and narrow therapeutic windows^9^.

T cell based immunotherapies have radically transformed the treatment of cancers, often overcoming a lack of T cell infiltration in tumours^10^. The Bispecific T cell engagers (BiTE), with their abilities to bind simultaneously on one arm to a target tumour antigen and another to CD3 expressed on T cells, can redirect T cells to induce tumour lysis. Enhanced treatment efficacies have been achieved with many BiTE antibodies like the FDA approved Blinatumomab for the treatment of B cell malignancies^11^. Currently, RON antibodies have never been pursued in a bispecific T cell engager format.

We have previously reported a panel of mouse monoclonal antibodies to the unglycosylated extra cellular domain of RON and determined that they recognize cryptic epitopes which are present on intracellular forms of RON but are not exposed at the cell surface of cancer cells grown in tissue culture^12^. Surprisingly, we found that one of these antibodies, 6E6 which recognizes a sulfhydryl bond constrained epitope, to be highly effective in xenograft models despite the lack of apparent surface expression. Here we extend these studies by creating a second panel of antibodies directed against the glycosylated extra cellular domain of RON obtained by mammalian expression. The change of form of the antigen resulted in antibodies that exclusively recognized denaturation sensitive epitopes. Some of the new antibodies show remarkable affinity for RON, determined using a kinetic exclusion assay, and bind tightly to the surface expressed form of the protein. Some antibodies in the panel act as potent antagonists blocking MSP signaling. All of the antibodies could be cloned and expressed as recombinant proteins and demonstrated complete specificity for RON showing no reaction with three different lines of RON knock out tumour cells produced by CRISPR. To assess the suitability for further development of the novel antibodies we cloned the antibody genes and reformatted them as human mouse chimeric antibodies and analysed their activity in antibody dependent cell killing assays compared to an approved antibody therapeutic. To further explore the potential utility of the new reagents we also labelled the two most promising antibodies with with 89-Zirconium (^89^Zr) and looked at their in vivo ability to image RON positive xenografts. We also reformatted the highest affinity antibody as a bispecific T cell engager and found very potent killing of RON tumor cells by redirected human T cells in vitro. This approach of early engineering of new antibodies promises to speed up the development of new clinical candidates by establishing the suitability of the selected V regions for further studies.

## Results

### Screening and characterization of antibodies targeting membrane bound RON

The unusual behavior of the antibodies that we produced when we immunized mice with bacterially produced RON protein domains has been described previously^12^. Essentially, we found antibodies that worked exceptionally well in immunoblotting and immunohistochemistry applications but showed weak surface binding to live cells in flow cytometry. Treatment of cells with mild fixatives or inhibitors of glycosylation massively enhanced specific binding in flow cytometry. Despite the apparent cryptic nature of the epitopes recognized by these antibodies several of them showed activity in xenograft assays.

In an effort to understand these results in more detail, we compared the activity of the antibodies inenzyme-linked immunosorbent assay (ELISA) and surface plasmon resonance (SPR) assays directed towards our original immunogens and preparations of the ECD of RON expressed in, and purified from, mammalian cells. This protein extended from amino acid Gly25 to Thr 957 and contained an N terminal His-tag. Surprisingly, the antibodies only bound to these RON preparations weakly, if at all in either SPR or ELISA formats (Data not shown). To confirm that the correct protein had been purified the preparation was analyzed by protease digestion and mass spectrometry (MS). The results showed that the protein was of the correct sequence but peptides containing the N-X-S/T motif required for glycosylation were undetectable by MS. This suggested that the preparation was highly glycosylated. It was however bound by a polyclonal antibody to RON, suggesting that it displayed novel epitopes not detected by our existing panel of antibodies. We therefore set out to make a new panel of anti-RON monoclonal antibodies using the mammalian produced His-tagged human RON preparation as the immunogen. In order to select for novel antibodies, we used the immunogen in an ELISA assay to screen the mice after immunization and to screen the hybridoma fusion and supplemented this screening assay with the immunofluorescent staining of two human cancer cell lines that express RON (HCT116 and T47D) in a high throughput 96 well based assay using a In cell Analyzer (GE Healthcare). The unusual behavior of our first set of antibodies in failing to bind to the mammalian RON preparation motivated us to use CRISPR Cas9 to “knock-out” the RON gene from three cell lines (HCT116, HT29 and T47D cells) to use as controls in counter screening assays (Supplementary I).

**Figure 1:**
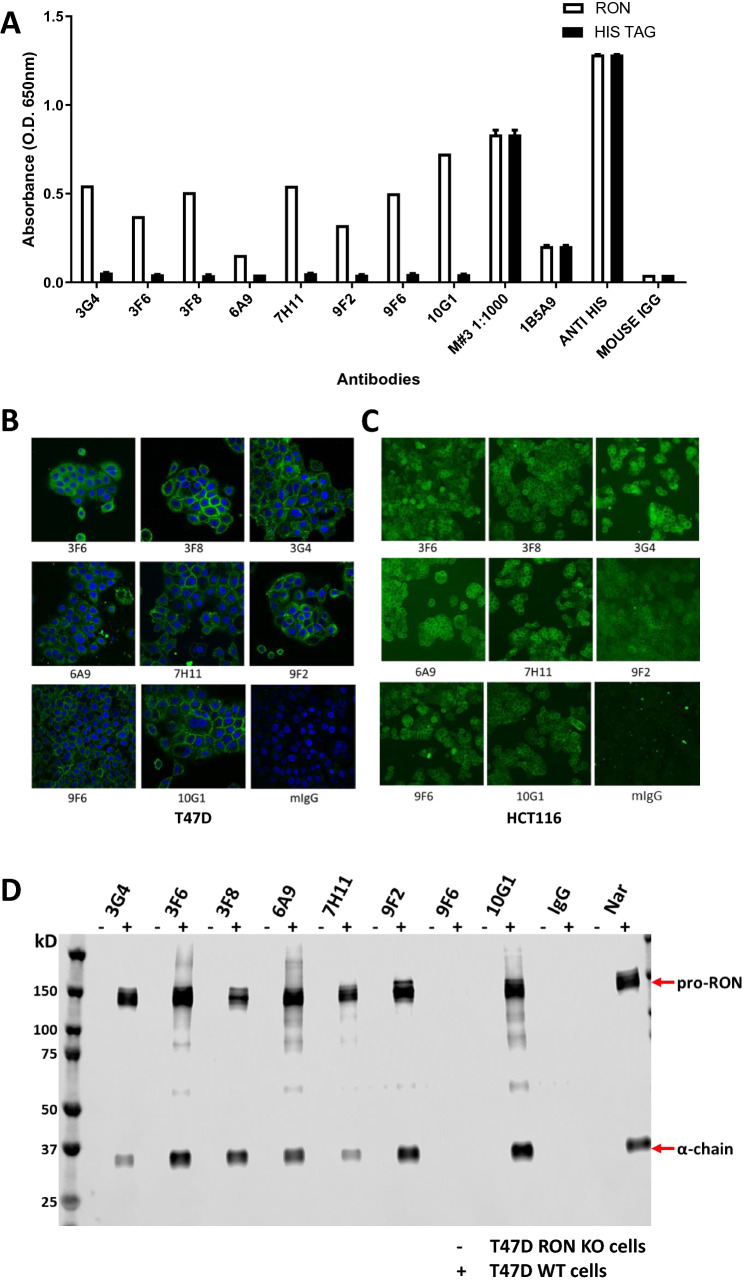

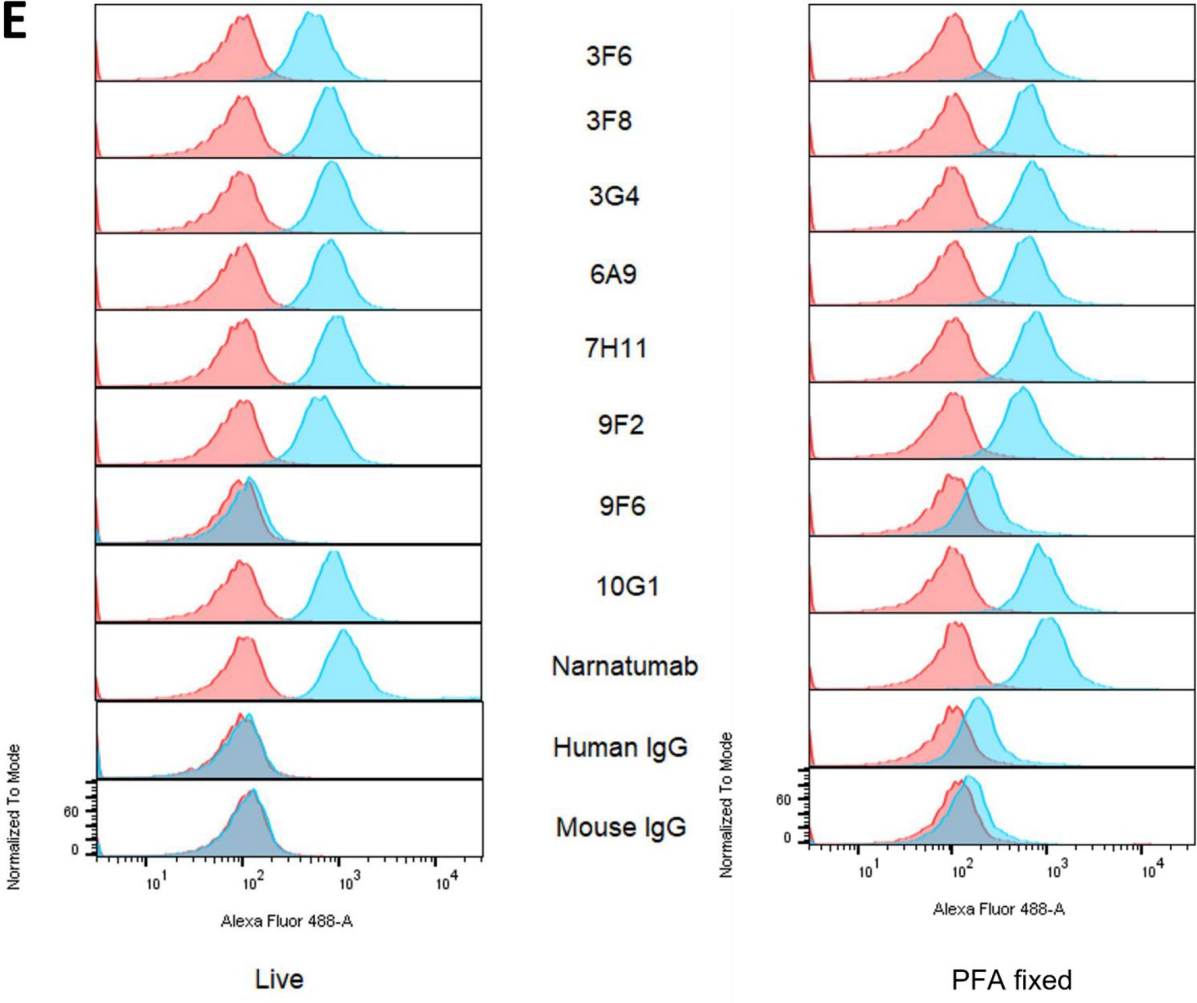
Screening and characterisation of RON antibodies via ELISA, cell staining, immunoprecipitation and flow cytometry.

Mice were immunized with the mammalian-produced RON protein in adjuvant, and mouse sera from one of the animals showed a high titre in the ELISA assay (Fig. 1A). In this assay we compared binding to the His-tagged RON protein to binding to another His-tagged protein. The commercial anti-RON antibody and the mouse serum from our immunized donor mouse showed binding to both proteins, as did a commercial antibody to the His-tag, however the eight selected new mouse monoclonal antibodies were shown to bind strongly to the mammalian cell produced His-tagged RON but not to the control His-tagged protein.

We next examined the binding of the antibodies to the cell surface of live unfixed human cancer cell lines T47D and HCT116. To block receptor mediated endocytosis, we fixed the cells with 4% paraformaldehyde after adding primary antibodies and washing off unbound antibodies before detecting bound antibodies with an Alex Fluor conjugated anti-mouse IgG antibody. All of the eight monoclonal antibodies showed strong cell surface staining by confocal microscopy, while a mouse IgG control gave only a weak background stain (Fig. 1B). As an additional control we used the CRISPR RON knockout cells in the same protocol and showed that none of the new antibodies could stain these cells, providing strong evidence that the antibodies were RON specific and devoid of non-specific cell surface binding (Supplementary II). We next tested the antibodies for their ability to immunoprecipitate the RON protein from cell extracts of the T47D tumor cell line. To probe the blot, we used the anti-RON alpha chain monoclonal antibody 6E6^12^ directly conjugated to peroxidase. This method gave extremely clean results. The control anti-RON antibody, Narnatumab, immunoprecipitated both the free alpha chain of RON at 35 kDa and uncleaved pro-RON at 150 kDa respectively. As expected, nothing was detected in the RON knock-out cell immune precipitates. Our new monoclonal antibodies showed strong and specific immunoprecipitation of the 150 kDa pro-RON and the 35 kDa RON alpha chain species, like Narnatumab.

Finally, we tested the antibodies’ ability to bind to cell surface RON using immunofluorescence (Fig. 1C,D) and flow cytometry (Fig. 1E). RON antibodies strongly stained the cell membrane in immunofluorescence. In our prior study we had found that the antibody 6E6 only bound to the cell surface after mild fixation with paraformaldehyde but could not bind to unfixed live cells. We therefore compared binding of the new panel of antibodies to both live cells and PFA fixed cells. In the live cell assay Narnatumab and seven of the eight new antibodies showed a clear completely right shifted population of strongly stained T47D cells (blue) but no staining in RON knockout T47D cells (lighter peak) (orange). No staining was observed with the new antibody 9F6 or with any of the isotype control or secondary only antibodies. Similarly, Narnatumab and all the new antibodies stained PFA-fixed cells, except for 9F6. As in the live cell assay none of the antibodies stained the PFA fixed isogenic T47D RON KO cells, suggesting that the antibodies were highly specific to RON.

### Determination of the binding affinities of the antibodies by KinEXa

**Figure 2:**
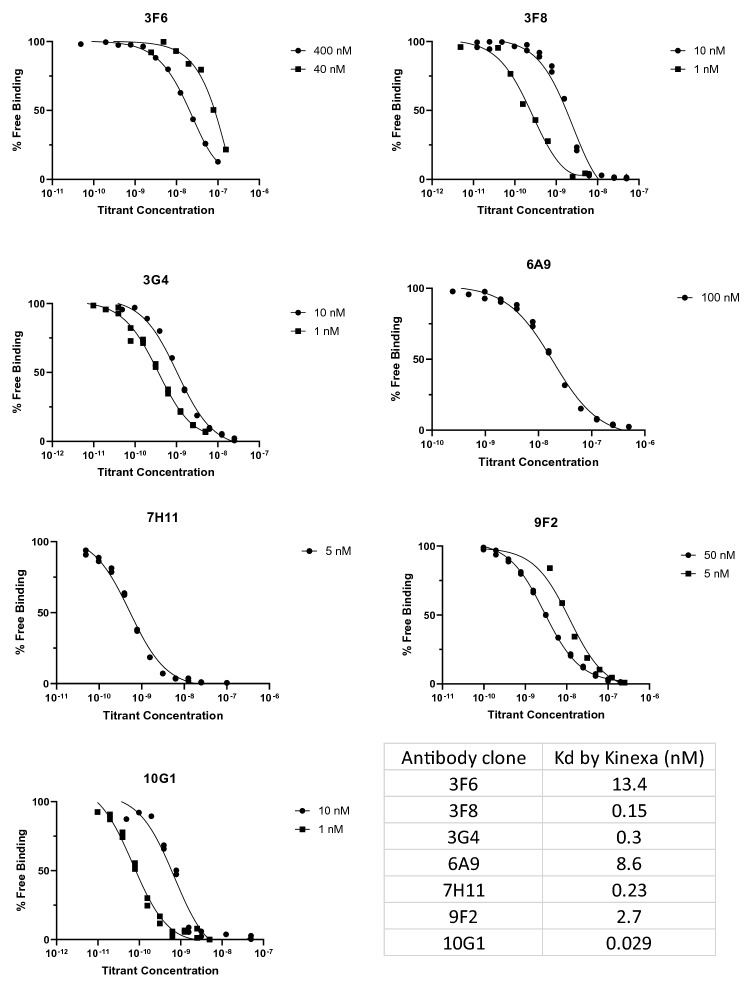
KineXa determined binding affinities of anti-RON antibodies on mammalian expressed extracellular RON antigens.

An initial analysis of the binding of the new antibodies using Surface Plasmon Resonance revealed that many of the new antibodies had exceptionally slow off rates making a true determination of their Kds problematic^13^. This limitation of SPR for highly avid antibodies has been described previously and the use of kinetic exclusion assay has been proposed to overcome this limitation allowing measurements in the sub nanomolar range. We employed the Kinetic Exclusion assay (KinEXa) method to measure the Kd values of all the new antibodies using a fixed concentration of the recombinant mammalian expressed protein as the target antigen and a twelve-point dilution curve of each antibody^13^. We dropped antibody 9F6 in this assay as it seemed less avid than the other antibodies in preliminary SPR binding assays. The remaining seven antibodies gave reproducible results in duplicate analysis (Fig. 2). The binding data and the resultant Kd values showed a wide range of affinities, with four (3F8, 3G4, 7H11 and 10G1) of the seven antibodies binding with sub-nanomolar affinities. The 10G1 antibody stood out with the exceptionally high binding affinity of 29 pM. It has been reported that other species produce higher affinity antibodies than mice. In particular, rabbits have been shown to do so. However, it is clear from our data that exceptionally avid antibodies can be isolated using conventional monoclonal methodologies.

### Extracellular RON antibodies blocks MSP induced downstream signaling pathways

**Figure 3:**
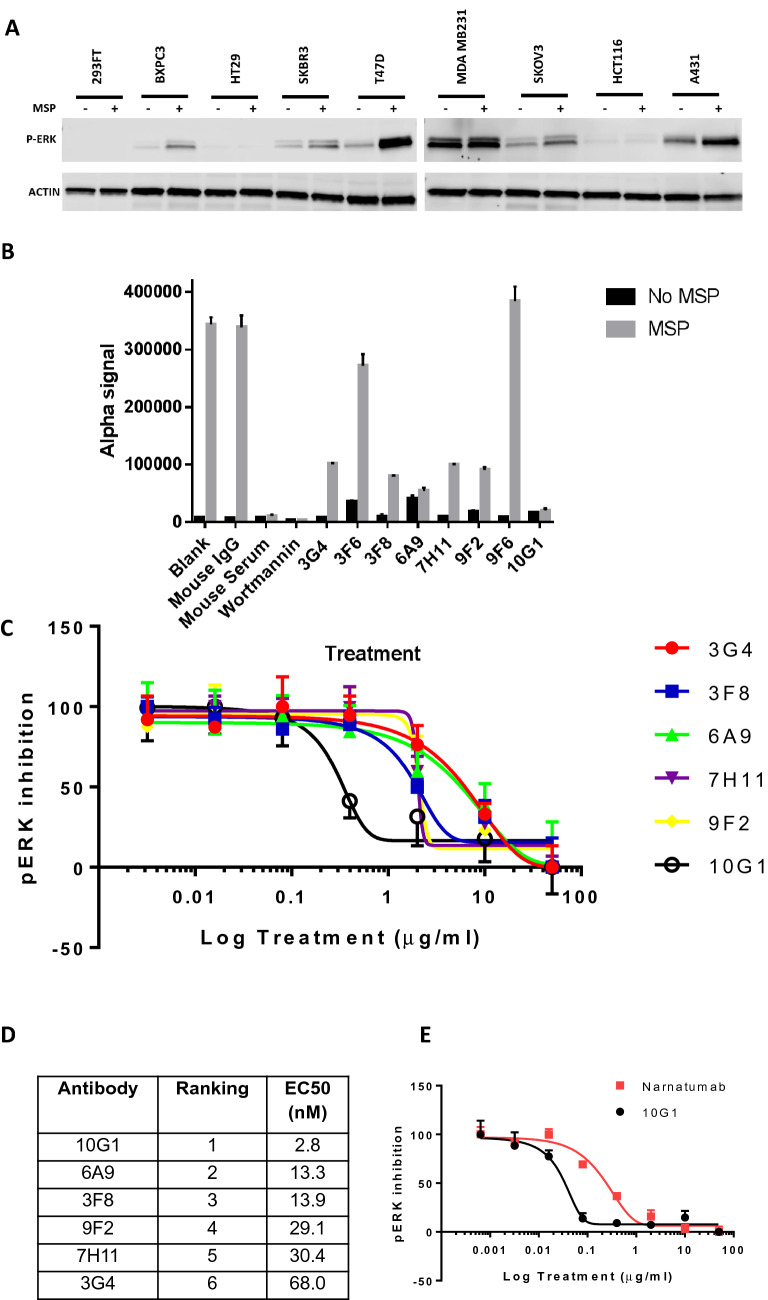
Anti-RON antibodies exert antagonositic effects on MSP induced increase in levels of phosphorylated ERK.

Next, we sought to determine if our new panel of antibodies to RON could inhibit the activation of the RON signaling pathway by MSP. First, we analyzed a panel of cell lines and studied the response to MSP by using immunoblotting for phosphorylated ERK 2 (Thr202/Tyr204) (Fig. 3A). While several of the cell lines responded to MSP stimulation with increased levels of phosphorylated ERK, the clearest signal was seen with the T47D cells line. Other cell lines showed weaker responses (HCT116 cells) or had high levels of ligand independent pERK (MDA MB231 cells). AlphaLISA assay (Perkin Elmer) was subsequently used to quantitatively measure the antibody-mediated inhibition of RON signaling upon MSP stimulation by measuring the levels of downstream phosphorylated ERK. While two of the antibodies (3F6 and 9F6) were not antagonistic (Fig. 3B), the remaining six antibodies showed dose-dependent inhibition of RON signalling (Fig. 3C), with IC50s ranging from 2.8 nM of 10G1 to 68 nM for 3G4 (Fig. 3D). In comparison, Narnatumab had an IC_50_ of 14.9 nM, nearly an order of magnitude less potent than 10G1 (Fig. 3E).

In our screening studies, western blot was performed on full length RON transfected 293FT cells or HCT116 cells expressing endogenous RON with our hybridoma supernatants as primary antibodies. However, none of the eight antibodies, unlike 6E6, were able to detect the RON bands on either RON transfected cell lysates or lysates expressing endogenous RON, implying that the antibodies are unable to detect the denatured form of RON produced in the immunoblotting method and detect only folded RON protein in applications like cell staining, ADCC or flow cytometry analyses (Table 1). This suggests that the epitope the antibodies bind to is lost upon denaturation.

**Table 1:** Summary table showing the characterization of anti-RON antibodies by binding assays.

Assay	Clone name							
	3G4	3F6	3F8	6A9	7H11	9F2	9F6	10G1
ELISA	+++	+++	+++	+++	+++	+++	++	+++
WB- Trans	−	−	−	−	−	−	−	−
WB- Endo	−	−	−	−	−	−	−	−
Cell staining- Trans	+++	+++	+++	+++	+++	+++	++	+++
Cell staining- Endo	+++ (Mem)	+++ (Mem)	+++ (Mem)	+++ (Mem)	+++ (Mem)	+++ (Mem)	+++ (Mem)	+++ (Mem)
Cell staining- RON KO	−	−	−	−	−	−	−	−
FACS (T47D cells)	++	++	++	++	++	++	++	++
IP	+++	+++	+++	+++	+++	+++	+++	+++

Trans stands for transfected cells, Endo stands for endogenous RON expressing cells, RON KO stands for RON knock-out cell HCT116 cells. Mem indicates membrane staining. IP stands for immunoprecipitation.

### RON antibodies exhibit therapeutic efficacy through ADCC

**Figure 4:**
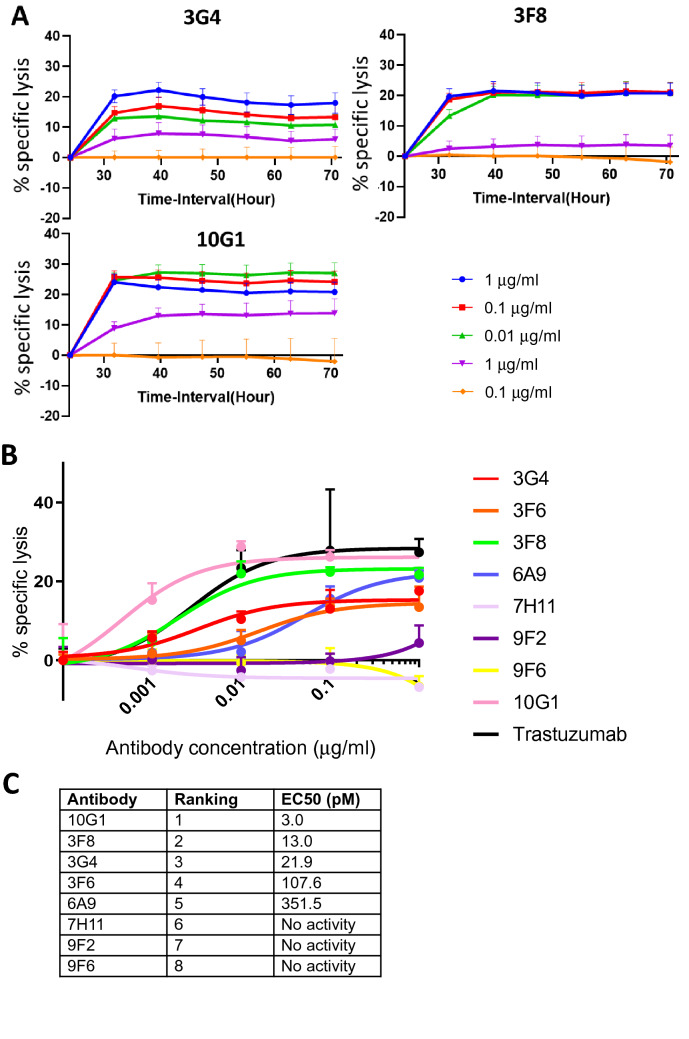
Antibody dependant cellular cytotoxicity activities of anti-RON antibodies tested in vitro.

One key mechanism by which therapeutic monoclonal antibodies can exert their effects is through the Fc dependent recruitment of immune effector cells that mediate antibody dependent cell cytotoxicity (ADCC). For this assay we used purified human NK cells and titrated the eight new antibodies over a wide range of concentrations (from 0.1 × 10^–12^ to 1 × 10^–6^ g/ml) using an impedance-based cell cytotoxicity assay (xCELLigence). The assay allows real time monitoring of target cell lysis after the addition of antibodies and effector cells. The plots show (Fig. 4B) that five of the antibodies showed ADCC activity, with dose dependent cell killing detectable at the first time point (40 min) but they varied greatly in their potency. Specific lysis data (Fig. 4A,B) were calculated from normalized cell index and baseline (no effector cells) subtracted curves using the xCELLigence RTCA data analysis software. We compared the antibodies to the clinically important anti-HER2 antibody Traztuzumab in this assay and found in keeping with literature that it had an EC_50_ of around 13 pM. Two of our antibodies (3F8 and 3G4) showed similar values to Traztuzumab while again the 10G1 antibody showed exceptional activity with an EC_50_ value of 3.0 pM (Fig. 4C).

### Radiolabeled RON antibodies are specifically localized in RON expressing tumours

The use of radiolabeled antibodies both for imaging as well as therapeutic radiopharmaceuticals is gaining increased interest. We labeled the 10G1 and 3F8 antibody directed to a surface exposed epitope with ^89^Zr after p-SCN-Bn-Deferoxamine (DFO)-conjugation. Both radiolabeled antibodies demonstrated a purity > 99% and displayed a significantly higher signal on RON positive (HT29) cells compared to RON negative (RON null HCT116) cells in vitro (Supplementary Fig. IIIA)*.*

**Figure 5:**
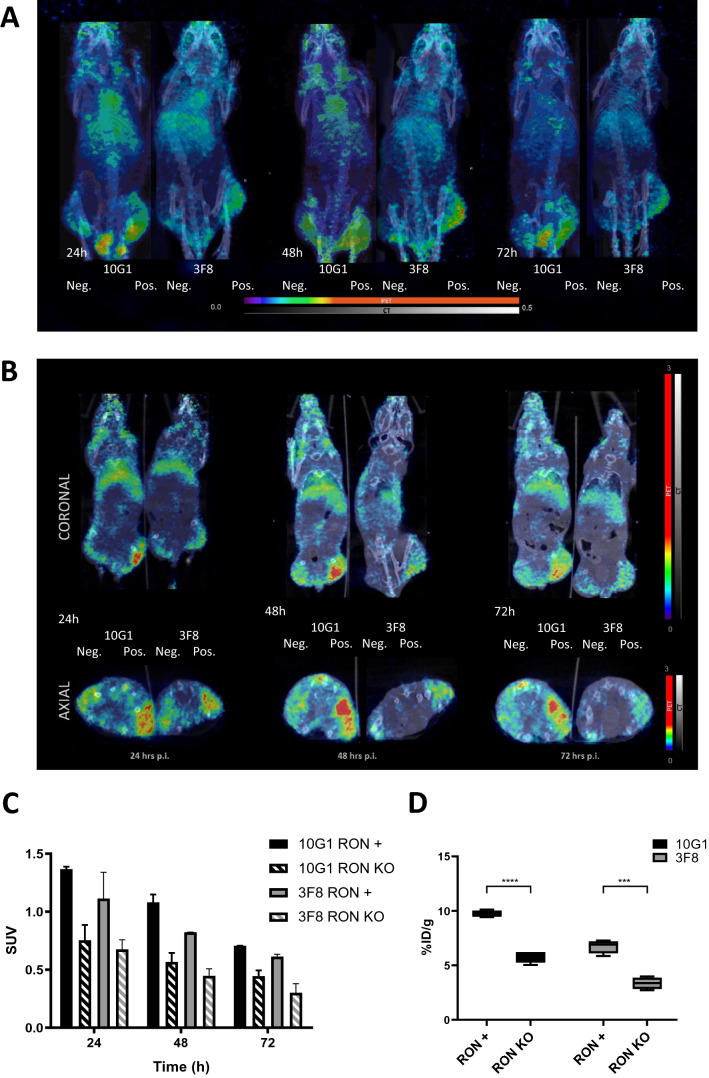
RON antibodies selectively target RON-positive HT29 xenografts.

For the in vivo study, tumours were established by injecting 5X10^6 HT29 cells on the right flank, and 5X10^6 HCT116 RON knockout cells on the left flank. PET imaging performed at 24, 48 and 72 h post injection, followed by ex vivo analysis of tumour uptake at 72 h post injection, showed selective accumulation of the ^89^Zr-labeled antibodies in the RON positive tumors, significantly higher than for the RON-negative tumors. This difference was still significant when corrected for tumor size (Fig. 5). There was no significant difference in tumour size between RON expressing and KO tumours at the imaging time point (72 h p.i.) for both radiolabelled Ab’s (Supplemental Fig. IIIC). After dissection no visible difference in vascularisation of the different xenografted tumours was observed. As expected for full-sized antibodies, activity was also present in blood and urine (Supplementary Fig. IIIB).

### Novel bispecific CD3-10G1 antibodies engaged T cells for specific killing of RON positive T47D cells

**Figure 6:**
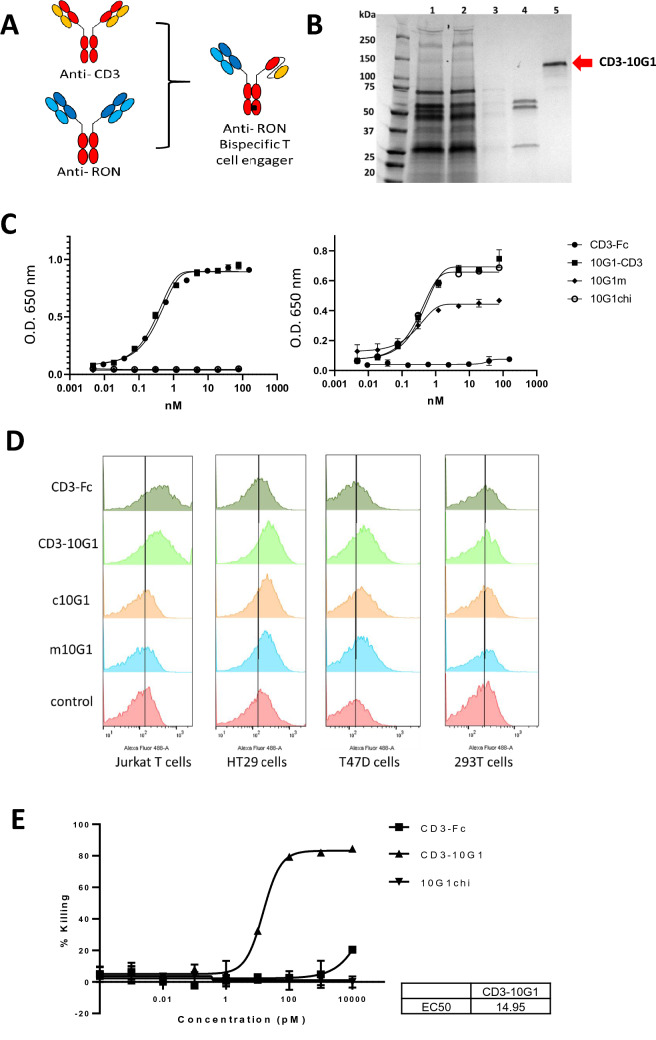
CD3-10G1 bispecific antibody engages T cells to kill T47D breast cancer cells in vitro.

10G1 was chosen to be our lead antibody for development into a bispecific T cell engager due to its superior binding affinity. A [1 + 1] bispecific antibody format was used where the anti-RON 10G1 Fab was joined to an anti-CD3 scFv via the knob and hole technology on the heavy chain Fc (Fig. 6A). CD3-10G1 were recombinantly expressed in ExpiCHO cells and purified as shown in Fig. 6B. In the reduced form, the antibody presents as a 25 kDa light chain, 50 kDa heavy chain and a 55 kDa scFv-Fc. In the non-reduced form, the antibody is approximately a 130 kDa molecule (Fig. 6B, Lanes 5–6). The purified CD3-10G1 was tested for functional binding activity in ELISA with coated human CD3, extracellular RON and control His tag antigens and confirmed to be specifically binding to CD3 and RON (Fig. 6C), with no cross reactivity to His tag protein. To confirm binding of CD3-10G1 to CD3 and RON positive cells, flow cytometry analyses was performed on CD3 positive Jurkat cells and RON positive T47D and HT29 cells. 293 T cells were used as a control to confirm non-binding. The bispecific CD3-10G1 bound to Jurkat cells, HT29 cells and T47D cells but not 293 T cells showing the retention of antibody specificities (Fig. 6D). A T cell killing assay was performed on Xcelligence to investigate if CD3-10G1 have cytotoxicity activities on RON positive T47D cells. T cell killing curves generated from Xcelligence showed that the CD3-10G1 antibody have superior T47D killing activities over isotype control and chimeric 10G1 antibody, with an IC50 of 14.95 pM (Fig. 6E).

## Discussion

### A new panel of antibodies to RON

While a vast array of recombinant methods has been devised to produce therapeutic antibodies, these are generally complex and expensive. The use of huge phage libraries has allowed the production of clinically remarkably successful antibodies such as Humira while the use of humanized mice has also been successful and produced antibodies like FDA approved anti-EGFR antibodies Cetuximab and Panitumumab^14,15^. More recently, B cell cloning using antigen selections has become effective^16^. However, despite these advances the traditional methods of hybridoma production are very well established and can take advantage of decades of experience in immunization protocols and screening methods^17^. Indeed, many of the best-selling therapeutic antibodies have been developed with this workflow, using recombinant methods to humanize the initial mouse antibodies and improve their biophysical properties^18^. Most recently the use of antibody drug conjugates, bi-specific antibodies, and recycling antibodies, along with the development of the CAR-T cells has enormously enhanced the potential of developing tumour specific immunotherapies^19,20^. Here we used chimeric and bispecific antibody constructs and ^89^Zr-labelling followed by PET imaging to further validate antibodies that showed high affinity, functional antagonism and potent ADCC activity.

Receptor tyrosine kinases overexpressed and mutated in cancer cells have proven to be exceptionally good targets for such approaches and antibodies to the EGFR and HER2 receptors have proved very useful in the treatment of breast cancer and colon cancer^21^. However, to date, no successful clinical antibodies to the related Met and RON receptors have been produced^2^. The reasons for failure can be many, one of the reasons might be that RON is alternatively spliced into many isoforms and depending on the epitope^2^, the antibody might miss out on targeting the RON molecule. To capture as many isoforms as possible, we designed our antibody to target the alpha chain of RON, missing out on only the short form RON as a limitation. Another possible reason is that tested molecules were only tried as simple antibodies, instead of modalities like drug conjugates or recycling antibodies. As such, their efficacies depend solely on signaling blockade, induction of the complement cascade, or Fc-mediated recruitment of NK cells or macrophages and was insufficient for complete reduction of tumour growth^22^. To increase the efficacies for RON targeting agents, more potent methods must be employed^5^. Here we generate a panel of new high affinity antibodies specific to RON. In particular, the 10G1 antibody binds with picomolar affinity (Kd) and is active in ADCC assays at single digit picomolar concentrations. 10G1 also performs exceptionally well as an in vivo imaging agent and we report the novel development of 10G1 as a bispecific T cell engager as a T cell therapeutic with improved anti-tumour efficacies.

### RON antibodies display profiles suitable for clinical development as cancer therapeutics

The cost of bringing an antibody molecule from ‘bench to bedside’ is remarkable. A study done by collecting data from 13 big pharmaceutical companies estimated the cost of development of one new biologic molecule to be around $1.8 billion USD^23^. As such, it is critical that most effort should be put into the selection of lead antibody candidates at the early discovery stage, to ensure the highest success rate for the antibody to reach the clinic^24^. Despite the initial promising biological functions shown by RON targeting antibody Narnatumab in preclinical models, the failure of the molecule when tested in phase I clinical trials was partially attributed to the molecule’s poor biophysical properties^4^. Lead monoclonal antibody candidates for drug development are typically selected for their high affinities and specificities to the target, potency and biological activities, and abilities to evoke Fc receptor functions^25^. From our panel of new anti-RON antibodies, our antibody with the highest binding affinity of 2.9 pM. 10G1 was chosen as the lead candidate for its ability to specifically bind to and immunoprecipitate RON expressed on the surface of cancer cells, potently block MSP stimulated downstream signaling of RON receptor, and its ability to elicit strong antibody dependent cellular cytotoxicity (ADCC) responses which can aid in tumour cell elimination. Moreover, the antibody can be recombinantly expressed and purified at a higher yield than Narnatumab, and is stable and soluble at high concentrations, without the biophysical problems of Narnatumab.

### Zirconium radiolabeled-10G1 and 3F8 can be developed into companion diagnostics for clinical imaging of RON positive tumours

The use of radiolabeled antibodies for the non-invasive diagnostic and detection of tumours biomarkers on primary tumours and metastases across different tumour types has been long established since the 1980s^26^. In the present study, we demonstrate that the antibodies can be successfully radiolabelled with ^89^Zr with high purity and retained antigen-specific binding both in vitro and in vivo (Fig. 5, Supplemental). Our in vivo distribution studies using ^89^Zr-10G1 and ^89^Zr-3F8 showed that the antibodies were able to specifically colocalize and accumulate in the xenograft tumour expressing RON with little cross reactivity to the RON knockout tumour. Whereas the antibodies demonstrated similar profiles in general, ^89^Zr-10G1 demonstrated higher tumour uptake and a slower blood clearance compared to ^89^Zr-3F8. The radioactivity levels in the urine were in the same range for both radioactively labeled antibodies 72 h p.i. Even though our study demonstrated promising data with the antibodies as in a whole IgG format, a potential future improvement could be to explore their use as immunoPET agents as smaller antibody fragments like a minibody, scFv or Fab fragment to potentially improve tumour penetration and reduce the blood circulation time, allowing for a higher tumour to blood ratio and a shorter duration for maximum contrast imaging^27,28^. As clinical research in oncology moves towards precision medicine, the development of immunoPET agents like 10G1 as companion diagnostics is critical to aid in the achievement of this goal, allowing for an improvement in the method for diagnosing RON related malignancies in the clinic, especially in cases where pathological results are not able to confirm and identify true positive disease. Here, immunoPET imaging agents can often quantitatively identify and measure the expression of the targeted antigen, predicting accurately the patient’s response to anti-RON antibody treatment.

### The nature of epitopes recognised by antibodies to RON and the impact of the immunogen on epitope selection

Our studies on antibodies to RON have a general significance in antibody development to the extracellular domain of tyrosine kinase receptors for diagnosis, therapy and imaging. Perhaps the most striking observation has been the profound effect the immunogen has on the types of antibodies produced. The bacterially produced antigen^12^ and the mammalian cell produced antigen have both induced useful and highly specific antibodies. The antibodies to the bacterial produced antigen work very well in immunoprecipitation, immunohistochemistry and in immunoblotting. Their epitopes can be precisely mapped using synthetic peptide libraries or phage peptide libraries. They react strongly to intracellular forms of RON and can be used in flow cytometry of fixed cells. Their reaction to the RON protein expressed at the surface of cancer cells is weak however and is induced by treatment with tunicamycin^12^. The new antibodies described here show that this weak surface staining is not a feature of all antibodies to RON as the new antibodies, produced in response to the mammalian protein immunogen, show strong cell surface binding. They are highly effective in inhibiting MSP driven signalling and in the activation of ADCC with human donor NK cells. The epitopes of the new antibodies could not be mapped by phage peptide libraries or synthetic peptide libraries and the antibodies are unable to detect antigen by immunoblotting being directed to conformational epitopes. The immunogen thus defines the dominant epitopes recognised. While glycosylation of the mammalian produced RON protein is probably a major factor in this selection the panel of antibodies reveals unexpected features of RON expression in cancer cells since antibodies to the bacterially produced protein proved highly active in xenograft models. These two complementing panels of antibodies will permit a more thorough understanding of RON epitope exposure in human cancers through their use as imaging agents and in proteomic analysis of intracellular and extracellular forms of the RON protein. As has been the case with the EGFR protein and its therapeutic antibodies targeting different epitopes^29^, it may thus be possible to develop antibody therapies that are specifically directed against cancer cell expressed versus immune suppressor cell expressed forms of RON extending the value of RON as a target in the immunotherapy of cancer and permitting image guided personalised medicine.

### Anti RON 10G1 as a diagnostic-therapeutic antibody pair

Traditionally, in clinics, antibodies that are used for diagnostic purposes on immunohistochemistry tissue sections are limited for that use. Here, we propose a safer, less invasive, more effective and personalized alternative in proposing for the same antibody to be used as a companion diagnostic- therapeutic pair. Using 10G1 as an immunoPET diagnostic companion for imaging of RON positive tumours in patients, the need for an additional patient selection step will be eliminated as the imaging data can help to predict antigen expression and hence, response to the therapeutic antibody with almost 100% accuracy^30^.

RON is a highly pursued target for cancer therapeutics for its well documented involvement in cancer parthenogenesis and development. Antibody engineering methods have rapidly revolutionized the potencies of therapeutic anti-cancer antibodies in the past 20 years, including the use of new technologies like bispecific t cell engagers, antibody drug conjugates, and CAR T cells. With additional potencies there is always a risk of additional toxicities as RON is still expressed, albeit at low levels, in healthy tissues. In the case of a bispecific T cell engager of RON, there is also a possibility of a cytokine release syndrome due to on target off tumour targeting. However, from the successes of previously reported RON-ADCs with little and reversible toxicities, it instills confidence that RON is a very safe to target molecule for cancer therapy^5^, and that our bispecific antibodies will likely do very well in enhancing 10G1’s cytotoxic abilities with little irreversible toxicities using current antibody engineering efforts like mutating the Fc for the CD3-bispecific antibodies to silence Fc binding to FcγR and C1q on immune cells^31^. From our in vitro T cell killing assays with 10G1 bispecific T cell engager, the molecule shows huge promise for pharmaceutical development as it has greatly improved tumour killing abilities compared to the naked antibody. Combining strategies of having 10G1 as a diagnostic-therapeutic antibody pair and improving the therapeutic efficacy of 10G1 by developing it as a bispecific T cell engager, RON expressing cancers can be more effectively targeted in the clinics.

## Methods and materials

### Generation of mouse monoclonal antibodies specific to mammalian extracellular RON

ExpiCHO cells were used for expression of extracellular human RON protein from amino acids Gly25 to Thr957 fused to an N-terminal His-tag. The expressed protein was purified by Nickel affinity purification using fast purification liquid chromatography (FPLC) and used as an immunogen, following an optimized mouse immunization schedule^32^. Immunized mice were regularly checked for antibody titer against RON immunogen and a chosen mouse was sacrificed for hybridoma fusion. Cell supernatants from fused hybridoma cells were screened in ELISA against the immunogen and control His-tag protein for detection of specific binders.

### Immunofluorescence staining of RON binders in live cancer cells expressing endogenous RON

Breast cancer cell line T47D (ATCC Cat# HTB-133, RRID:CVCL_0553) and colorectal cancer cell line HCT116 (ATCC Cat# CCL-247, RRID:CVCL_0291**)** expressing endogenous RON were seeded in individual wells in a 96 well plate and allowed to adhere overnight. Cell culture supernatants from hybridoma cells were applied as primary antibodies for an hour following fixation by 4% paraformaldehyde. IgGs were detected with Alexa Fluor 488 conjugated anti-mouse IgG. Cells were counterstained with DAPI and viewed with the In cell Analyzer (GE Healthcare).

### Western blot analysis of RON antibody clones in cell lysates expressing endogenous and transfected RON

Cells were harvested and lysed by sonication in 0.1% Triton X PBS supplemented with protease inhibitor cocktail (Roche). The QuickStart Bradford protein assay (BioRad) was used to determine protein concentration. BSA was used as a protein standard. 20 µg of cell lysates were mixed with NuPAGE lithium dodecyl sulphate (LDS) and sample reducing buffer (Thermo Scientific), heated for 5 min at 95 °C and loaded into 4–12% Mini-PROTEAN precast gels (Biorad) for electrophoresis. Separated cell lysates were transferred onto nitrocellulose membranes using the Trans-Blot turbo transfer system device (Biorad). Blocking was performed with 5% milk or bovine serum albumin (BSA) in PBS supplemented with 0.1% tween (TBST). Hybridoma supernatants were applied to individual cell lysate strips as primary antibody and detected with goat anti-mouse IgG (H + L) (Jackson Laboratories). The enhanced chemiluminescence (ECL) reagent used was SuperSignal West Dura Extended Duration Substrate (Thermo Scientific, #34076). Imaging and acquisition were performed with Licor Odyssey Fc and Image Studio version 5.2.5 (https://www.licor.com/bio/empiria-studio/).

### Generation of RON knockout cell lines in HCT116, HT29 and T47D by CRISPR Cas9

HCT116, HT29 (ATCC Cat# HTB-38, RRID:CVCL_0320) and T47D cells were chosen for knockout of *RON MST1R* by CRISPR. A guide RNA (RRID:Addgene_101731) containing the spacer (GGCGGGAGGAGCTCCATCG) that directs Cas9 to cut at the ATG initiation codon of *MST1R* was cloned into pX458, a plasmid, which contains the gRNA scaffold, spCas9-3xNLS and an EGFP reporter. This plasmid was transfected into HT29 and T47D cells using Lipofectamine 3000, and EGFP-positive cells were selected by FACS. Single clones were isolated and screened for *MST1R* knockout by directed Sanger sequencing near the Cas9 cut site with the following primers (Forward: ggtccgctatcttggggc; Reverse: ctgggcaccacgtacttcac).

### Immunoprecipitation of RON with RON antibodies

1 × 10^7^ cells were harvested from T47D wildtype and T47D RON KO cell lines using RIPA buffer. 10 µg/ml of purified antibodies were added to 200 µg of cell lysate and allowed to bind overnight at 4 °C in individual tubes. Antibody-RON complexes were picked up using Protein G Dynabeads magnetic beads (Thermo Scientific) and eluted in 20 mM Tris–Glycine pH2.7 buffer. The eluted proteins were detected in western blotting using hydrogen peroxidase enzyme conjugated in-house anti-alpha RON antibody 6E6. SuperSignal West Dura Extended Duration Substrate (Thermo Scientific) was used for detection of chemiluminescence signal. Imaging and acquisition was performed using Licor Odyssey Fc and Image Studio (version 3.1).

### Flow cytometry analysis of RON antibodies with fixed and live cancer cells

1 × 10^6^ cells were harvested from T47D wildtype and T47D RON KO cell lines and washed in PBS. Cells were blocked and either paraformaldehyde fixed or stained live. Primary antibody staining was performed using 10 µg/ml of purified anti-RON antibodies. Cells were subsequently incubated with goat anti-mouse FITC-conjugated secondary antibodies (Invitrogen). FACS analysis was performed using FACS LSRII machine (Becton Dickinson). FlowJo (RRID:SCR_008520) (Tree Star Inc. USA) software was used for data analysis.

### Binding affinity studies of RON antibodies using Kinetic Exclusion Assay

Mammalian produced recombinant extracellular His-tag RON protein was used for affinity measurements. Affinity determinations were carried out in the fixed antigen format. Antibodies were titrated as two-fold dilutions into a fixed concentration of RON antigen. RON protein was detected using mouse monoclonal to 6xHis-Tag (Dylight@650, Thermo Fisher). All affinity measurements were carried out using the KinExa 4000 (Sapidyne Instruments).

### Study of downstream RON antibodies signaling using phosphorylation assays

60,000 T47D cells and T47D RON^-/-^ cells were seeded into individual wells in a 96 well plate. Varying concentrations of antibodies, wortmannin or control mouse sera were added to individual wells for an hour before addition of MSP (10 nM) for half an hour. Experiments were run in triplicate. The cells were harvested for p-ERK level analyses using the AlphaLISA SureFire Ultra p-ERK1/2 (Thr202/Tyr204) Assay Kit (Perkin Elmer) as per manufacturer’s protocol.

### Antibody dependent cellular cytotoxicity assay on RON antibodies

All antibodies used for the antibody dependent cellular cytotoxicity assay were carried out with engineered chimeric RON antibodies which retained the mouse Fabs with a human Fc backbone and expressed recombinantly. Use of the apheresis blood from healthy donors for this study was approved by the National University of Singapore Institutional Review Board (IRB reference number: 2017/2512). Written informed consent was obtained from donors and all experiments were performed in accordance to relevant guidelines and regulations. NK cells were isolated using the EasySep™ Direct Human NK Isolation Kit (Stemcell Technologies). The effector to target ratio used was 10:1 based on target T47D cells initial seeding. ADCC activities were measured using the xCelligence platform (Roche Applied Science) using plates with detector electrodes that quantify the number of cells attached to the bottom of the wells, reflected by a calculated cell index (CI). The CI was measured every 15 min over 72 h after the antibody treatment. Treatments were performed in triplicates, with averages and standard deviations calculated by the instrument.

### In vivo animal imaging studies on 3F8 and 10G1

#### Animal experiments

All animal experiments were performed in accordance with protocols approved by the Uppsala Committee of Animal Research Ethics (C33/16) and followed FELASA guidelines for animal welfare. This study is reported in accordance with ARRIVE guidelines.

#### In vivo xenograft model

Female nu/nu Balb/c mice (RRID:IMSR_ORNL:BALB/cRl) (*n* = 8) were housed under standard laboratory conditions and fed ad libitum. Tumor xenografts were formed by subcutaneous inoculation of approximately 1 × 10^6^ RON-positive HT29 cells on the right posterior leg and 1 × 10^6^ RON null HCT116 cells on the left posterior leg.

#### DFO-conjugation and ^89^Zr radiolabeling of 10G1 and 3F8

Conjugation of p-SCN-Bn-Deferoxamine (DFO) to Ab’s and ^89^Zr-labelling was performed as described previously^33^. In brief, Ab’s (2 mg/ml dissolved in 0.07 M borax buffer, pH 9.4) were incubated with the bifunctional chelator DFO (B-705, Macrocyclics Dallas, TX, USA) in the molar ratio of 1:3 (antibody to DFO) for 1 h at 37 °C using a thermomixer at 350 rpm. Unbound-DFO and Ab-DFO were separated with a NAP-5 column equilibrated with 0.25 M ammonium acetate (pH 5.4–5.6).

20 MBq ^89^Zr-oxalic (solid target production, clinical grade; kindly provided by Dr. Thuy Tran, KI, Stockholm) acid solution was added to 400 µg Ab at pH 6.8–7.2 (0.1 M Na_2_CO_3_ and 0.5 M HEPES were added for pH adjustment) and incubated for 1 h at room temperature while gently shaking at 350 rpm. Radiolabeling efficiency and radionuclidic purity was determined by chromatography strips (ITLC) using 0.2 M citric acid (pH 4.9–5.1) as mobile phase and analysis using a Fujifilm Bas-1800II phosphorimager (Fuji, Tokyo, Japan).

#### In vitro radioimmunoassay

1 nM of ^89^Zr-10G1 or ^89^Zr-3F8 was added to approximately 0.5*10^6^ HT29 (RON +) or HCT116 (RON KO) cells, and incubated at 37 °C, 5% CO_2_. After 24 h, cells were washed, trypsinized and counted. Cell-associated radioactivity was measured in a gamma counter (1480 Wizard 3″, Wallace, Turku, Finland). Radioactivity count was adjusted for cell number, and the signal on HT29 cells was normalized to HCT116 signal using GraphPad Prism 8 (RRID:SCR_002798) (GraphPad Software, San Diego, CA, USA). Statistical analysis of differences in uptake between antigen-positive and antigen-negative cells was performed using Graph Pad Prism 8 (RRID:SCR_002798), using unpaired student's t-test, with *p* < 0.05 (*), *p* < 0.01 (**), and *p* < 0.001 (***).

#### In vivo distribution of ^89^Zr-10G1 and ^89^Zr-3F8

Xenografted mice were injected via the tail vein with 50 µg of ^89^Zr-10G1 (n = 4) or ^89^Zr-3F8 (n = 4) antibodies (purity > 99%, injected activity 1.1 and 0.8 MBq), respectively. Whole-body PET/MRI/CT studies were performed under general anesthesia (sevoflurane 2.0–3.5% in 50% / 50% medical oxygen + air at 60 ml/ min) after 24 h, 48 h and 72 p.i. (i.v.) for ^89^Zr-10G1 (n = 2) and ^8^9Zr-3F8 (n = 2). Pre-injected mice were placed under sedation in the gantry of a small-animal nanoScan PET/MR scanner (Mediso Medical Imaging Systems Ltd., Hungary) and a whole-body PET scan was performed for 60 min in list mode followed by a CT scan in nanoScan SPECT/CT scanner (Mediso Medical Imaging Systems Ltd., Hungary) for 5 min. The breathing rate was monitored and animals were placed on the heated bed to prevent hypothermia. PET data was reconstructed into a static image and corrected for the time of injection using the Tera-Tomo ™ 3D reconstruction (6 subsets and 4 iterations). The raw CT data was reconstructed using filtered back projection. PET and CT dicom files were fused and analysed in PMOD 4.210 (PMOD technologies, Switzerland). Fused images, coronal and trans-axial, are presented in SUV scale (0–3).

Animals were euthanized 72 h p.i. with a mixture of ketamine and xylazine followed by heart puncture. Blood, urine (together with bladder) and tumours were then excised and weighed, and activity was measured in a gamma well-counter (1480 Wizard; Wallace Oy, Turku, Finland). Radioactivity uptake in the tissues was calculated as the percentage of injected dose per gram of tissue (%ID/g). Statistical analysis of differences in uptake between antigen-positive and antigen-negative tumors was performed with Graph Pad Prism 8 (GraphPad Software, San Diego, USA), using unpaired student's t-test, with *p* < 0.05 (*), *p* < 0.01 (**), *p* < 0.001 (***) and *p* < 0.0001 (****).

### Bispecific antibody T cell killing assay

10,000 T-47D cells (target cells) in 50ul complete RPMI per well were seeded into Xcelligence 96 well E-plates containing 50ul complete RPMI. PBMCs were obtained from commercially from Stem cell Technologies and isolated using EasySep Human T cell isolation kit. T cells were activated with CD3/CD28 antibodies and expanded using IL7/IL15 in vitro. Antibodies diluted in 50ul complete RPMI and 50,000 ex vivo expanded T cells (E:T ratio 5:1) in 50ul complete RPMI were added 24 h post seeding and the cell index was monitored over time. Cytolysis was calculated using:

% cytolysis = 100% × [(NCI of no antibody control)-(NCI of sample)]/(NCI of no antibody control), where NCI = normalized cell index.

## Supplementary Information

Supplementary Information.

## Data Availability

The data that support the findings of this study are available from the first author- Xin Yu Koh, (koh_xin_yu@imcb.a-star.edu.sg) upon reasonable request.
