# Supplementary material for: Conformation specific antagonistic high affinity antibodies to the RON receptor kinase for imaging and therapy
Source: Sci Rep. 2022 Dec 29;12:22564. doi: 10.1038/s41598-022-26404-7 (PMC9800565; doi:10.1038/s41598-022-26404-7)
Supplement: Supplementary file 1 — Supplementary Information. [file 41598_2022_26404_MOESM1_ESM.pptx]

## Slide 1
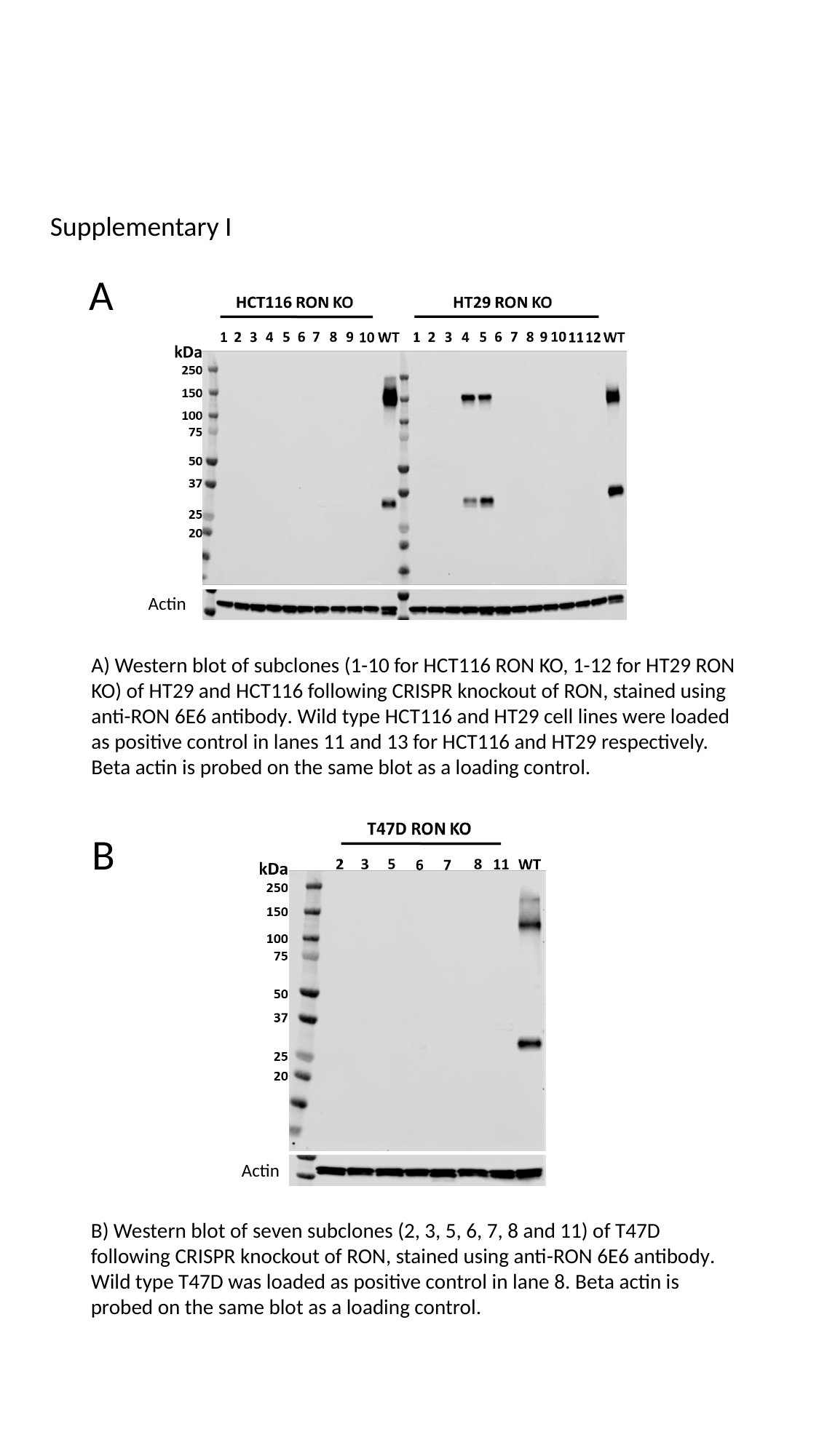

Supplementary I
A
Actin
A) Western blot of subclones (1-10 for HCT116 RON KO, 1-12 for HT29 RON KO) of HT29 and HCT116 following CRISPR knockout of RON, stained using anti-RON 6E6 antibody. Wild type HCT116 and HT29 cell lines were loaded as positive control in lanes 11 and 13 for HCT116 and HT29 respectively. Beta actin is probed on the same blot as a loading control.
B
Actin
B) Western blot of seven subclones (2, 3, 5, 6, 7, 8 and 11) of T47D following CRISPR knockout of RON, stained using anti-RON 6E6 antibody. Wild type T47D was loaded as positive control in lane 8. Beta actin is probed on the same blot as a loading control.

## Slide 2
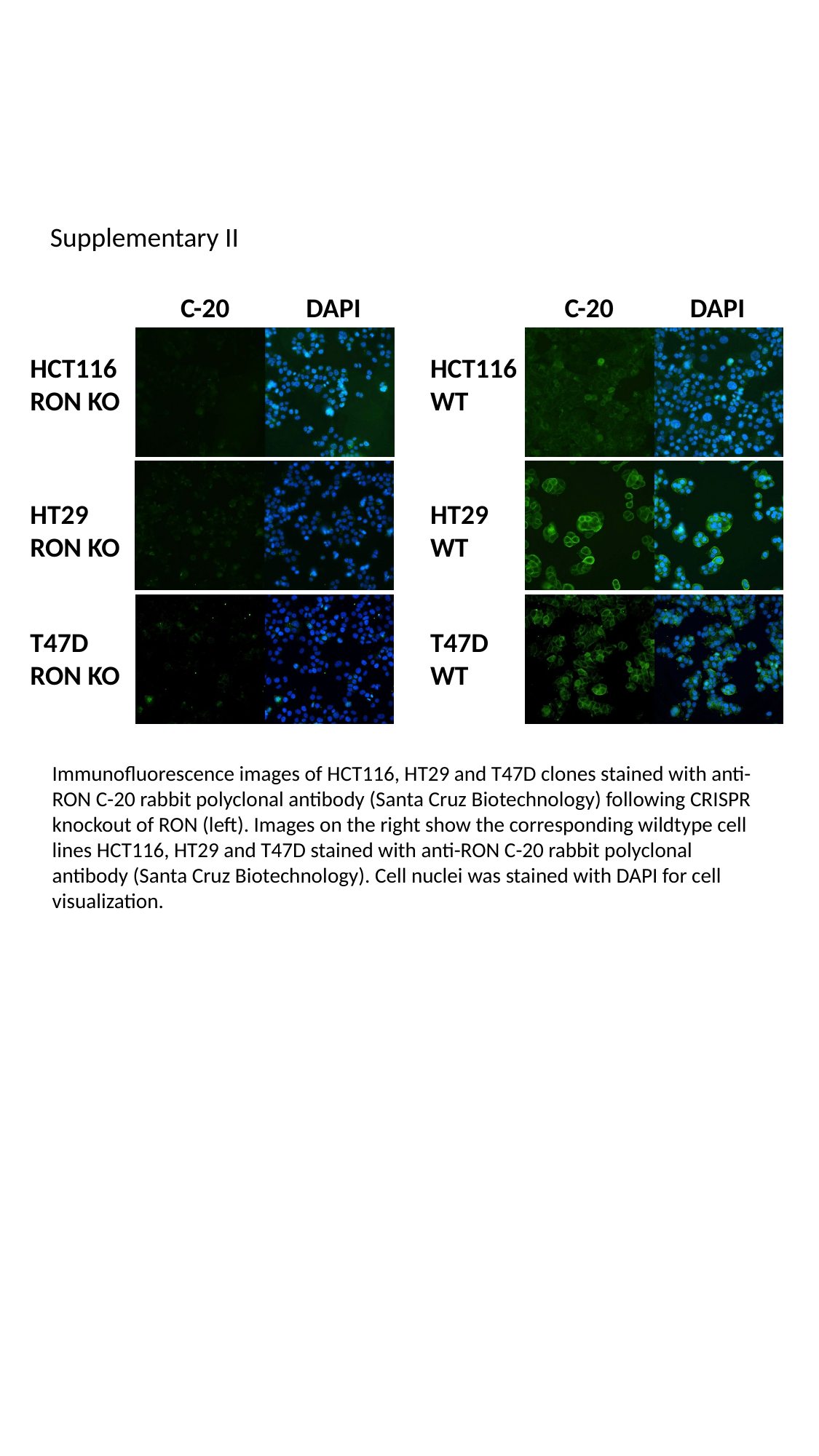

Supplementary II
C-20
DAPI
C-20
DAPI
HCT116 RON KO
HCT116WT
HT29 RON KO
HT29 WT
T47D RON KO
T47D WT
Immunofluorescence images of HCT116, HT29 and T47D clones stained with anti-RON C-20 rabbit polyclonal antibody (Santa Cruz Biotechnology) following CRISPR knockout of RON (left). Images on the right show the corresponding wildtype cell lines HCT116, HT29 and T47D stained with anti-RON C-20 rabbit polyclonal antibody (Santa Cruz Biotechnology). Cell nuclei was stained with DAPI for cell visualization.

## Slide 3
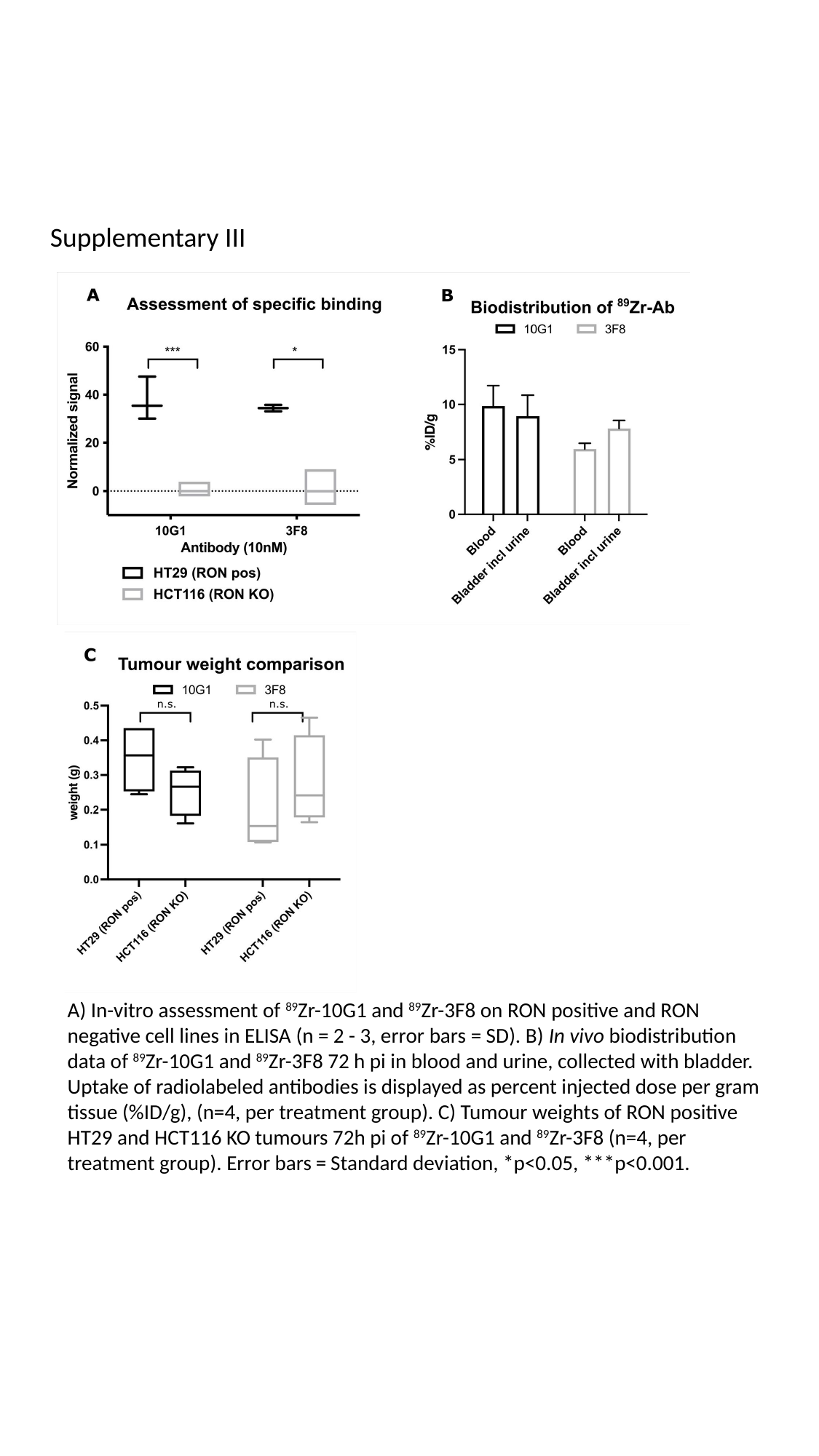

Supplementary III
A) In-vitro assessment of 89Zr-10G1 and 89Zr-3F8 on RON positive and RON negative cell lines in ELISA (n = 2 - 3, error bars = SD). B) In vivo biodistribution data of 89Zr-10G1 and 89Zr-3F8 72 h pi in blood and urine, collected with bladder. Uptake of radiolabeled antibodies is displayed as percent injected dose per gram tissue (%ID/g), (n=4, per treatment group). C) Tumour weights of RON positive HT29 and HCT116 KO tumours 72h pi of 89Zr-10G1 and 89Zr-3F8 (n=4, per treatment group). Error bars = Standard deviation, *p<0.05, ***p<0.001.

## Slide 4
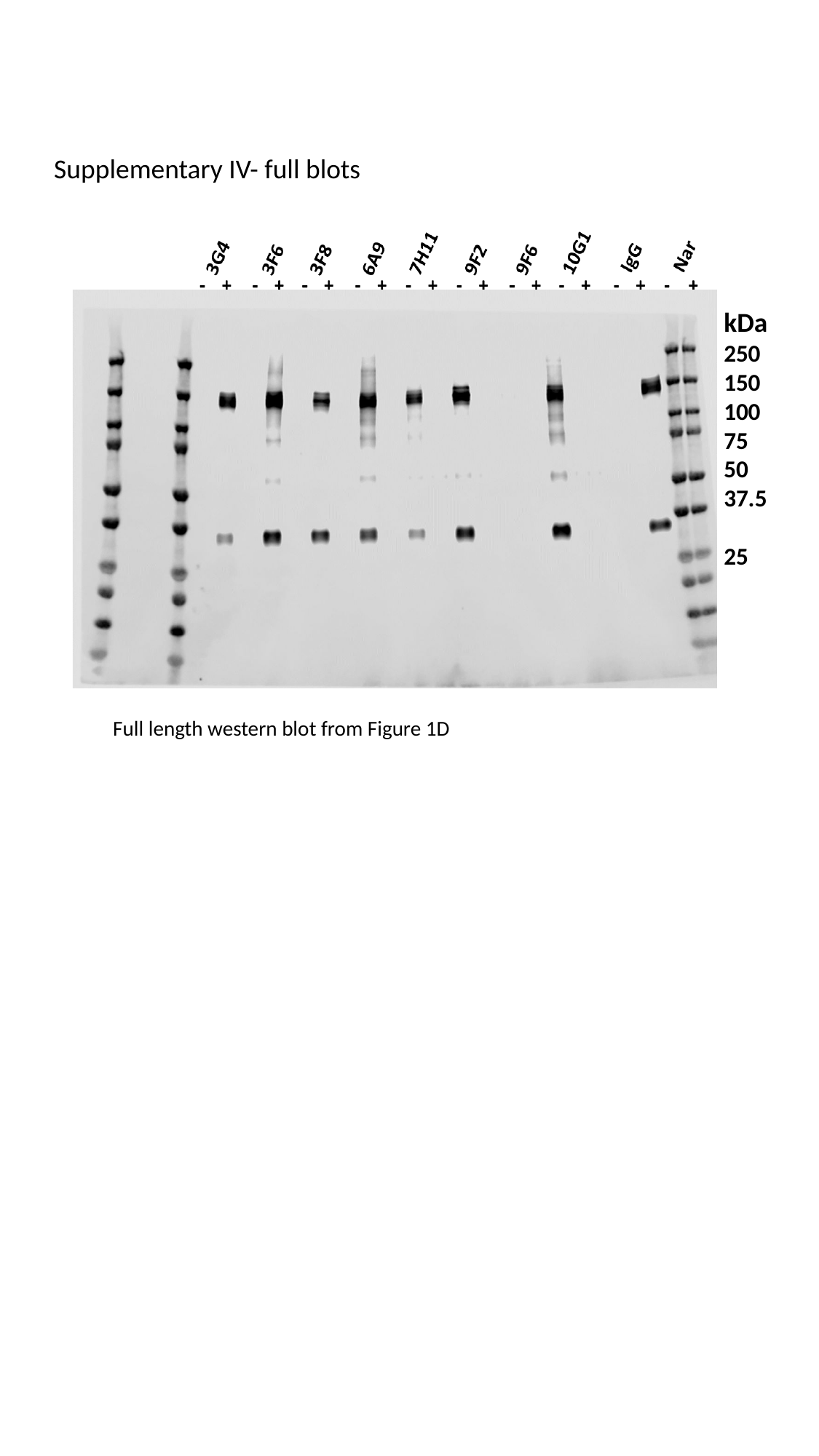

Supplementary IV- full blots
kDa
250
150
100
75
50
37.5
25
Full length western blot from Figure 1D

## Slide 5
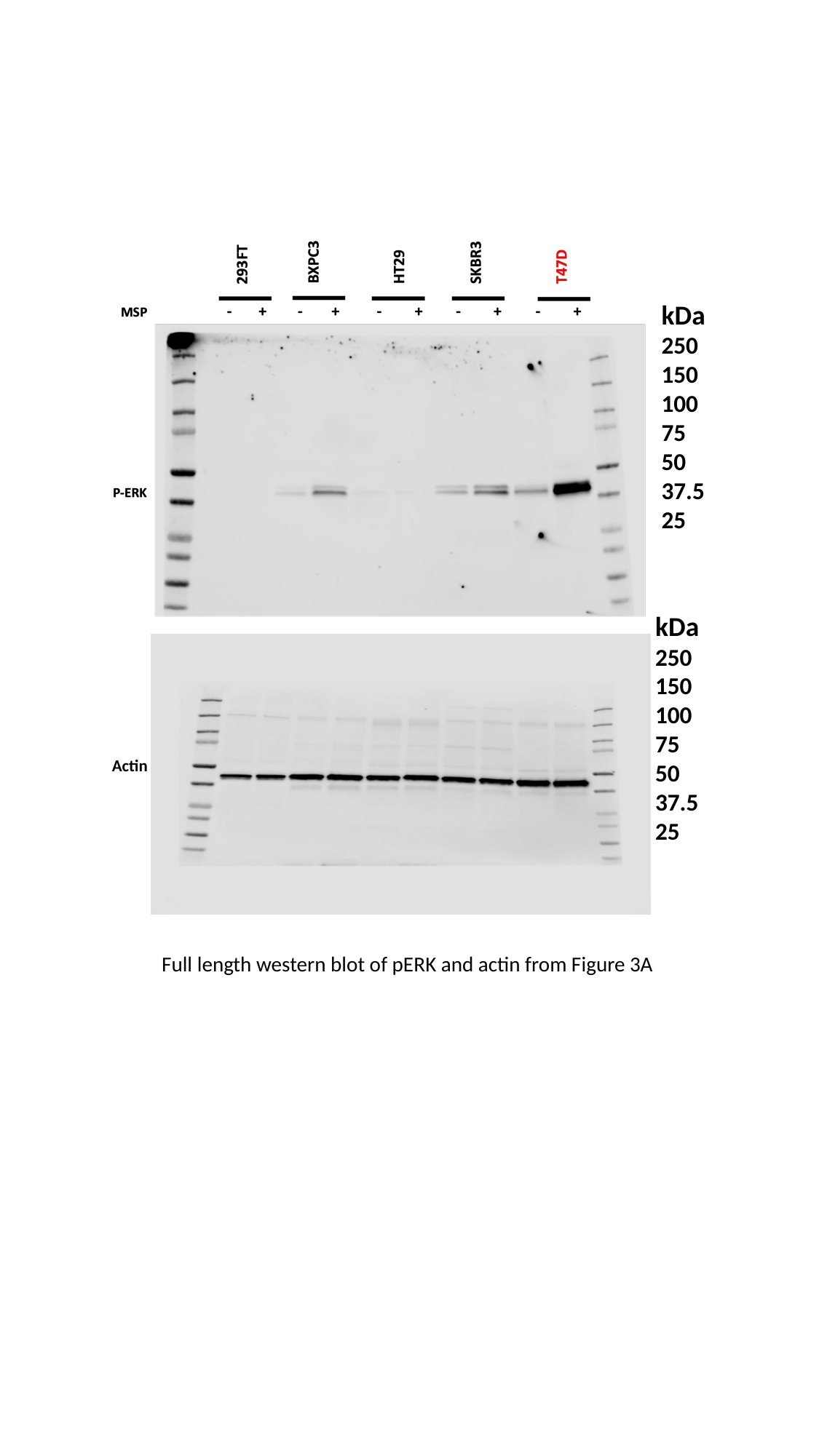

kDa
250
150
100
75
50
37.5
25
kDa
250
150
100
75
50
37.5
25
Actin
Full length western blot of pERK and actin from Figure 3A

## Slide 6
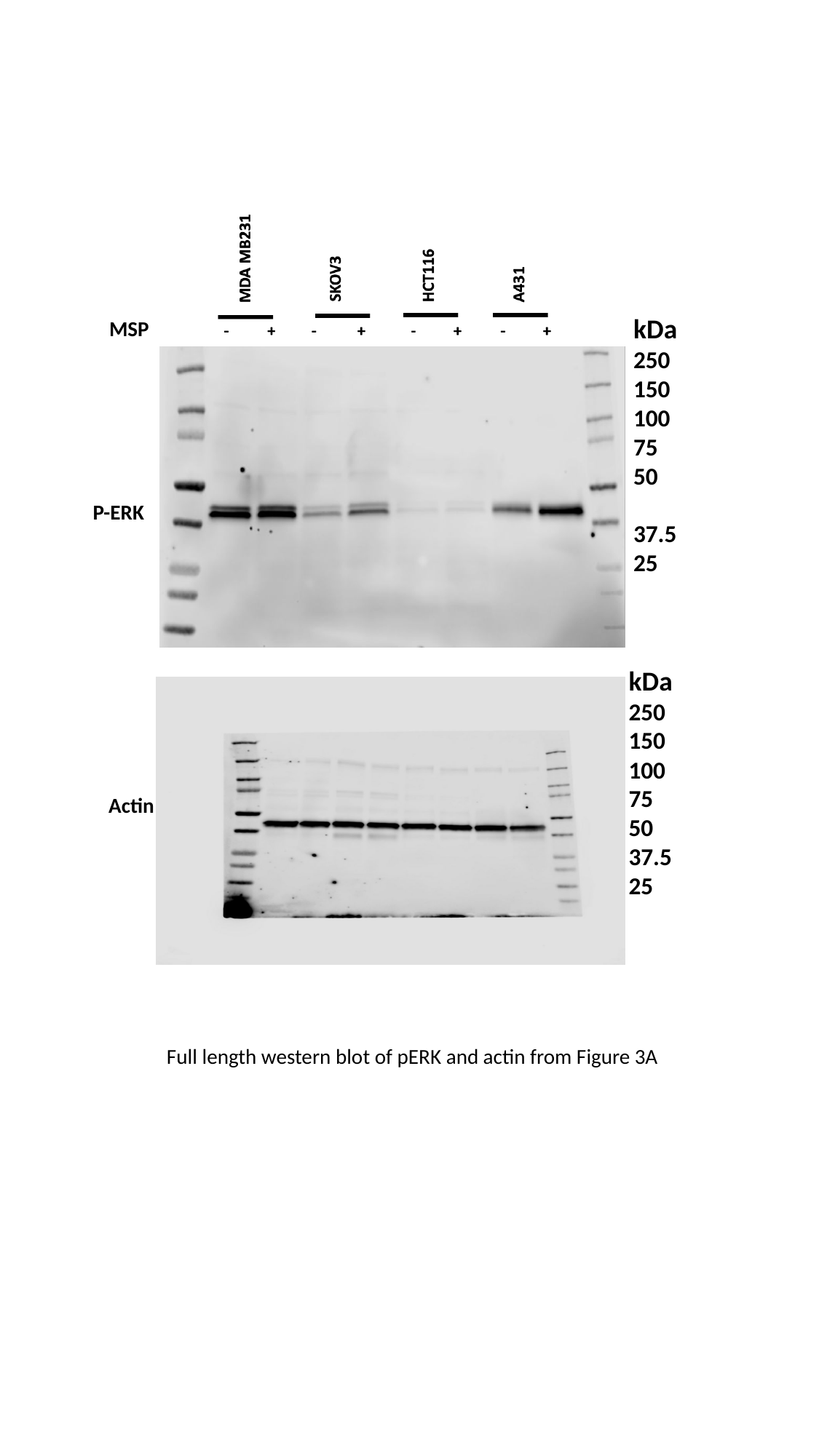

kDa
250
150
100
75
50
37.5
25
MSP
P-ERK
kDa
250
150
100
75
50
37.5
25
Actin
Full length western blot of pERK and actin from Figure 3A

## Slide 7
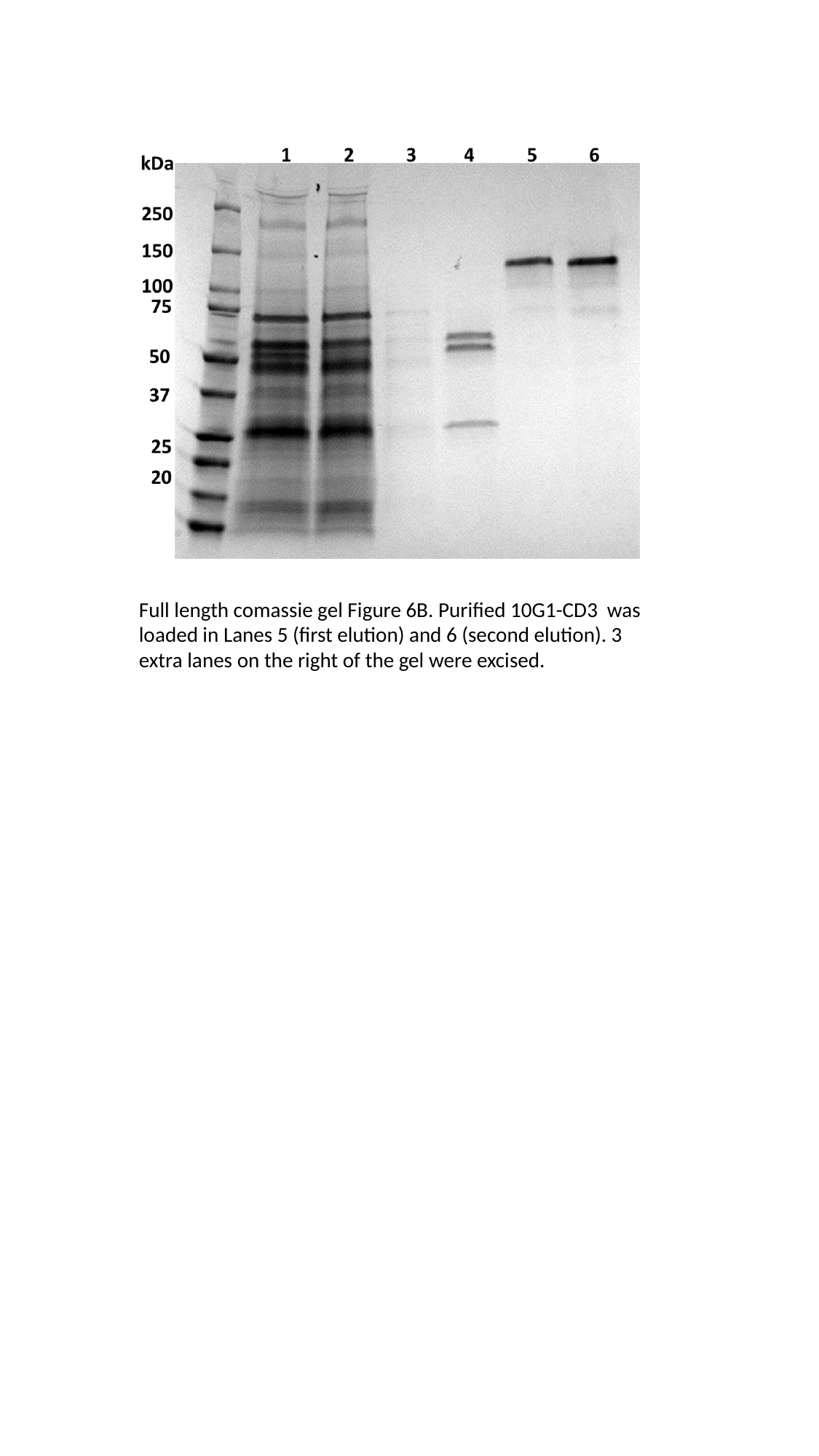

Full length comassie gel Figure 6B. Purified 10G1-CD3 was loaded in Lanes 5 (first elution) and 6 (second elution). 3 extra lanes on the right of the gel were excised.

## Slide 8
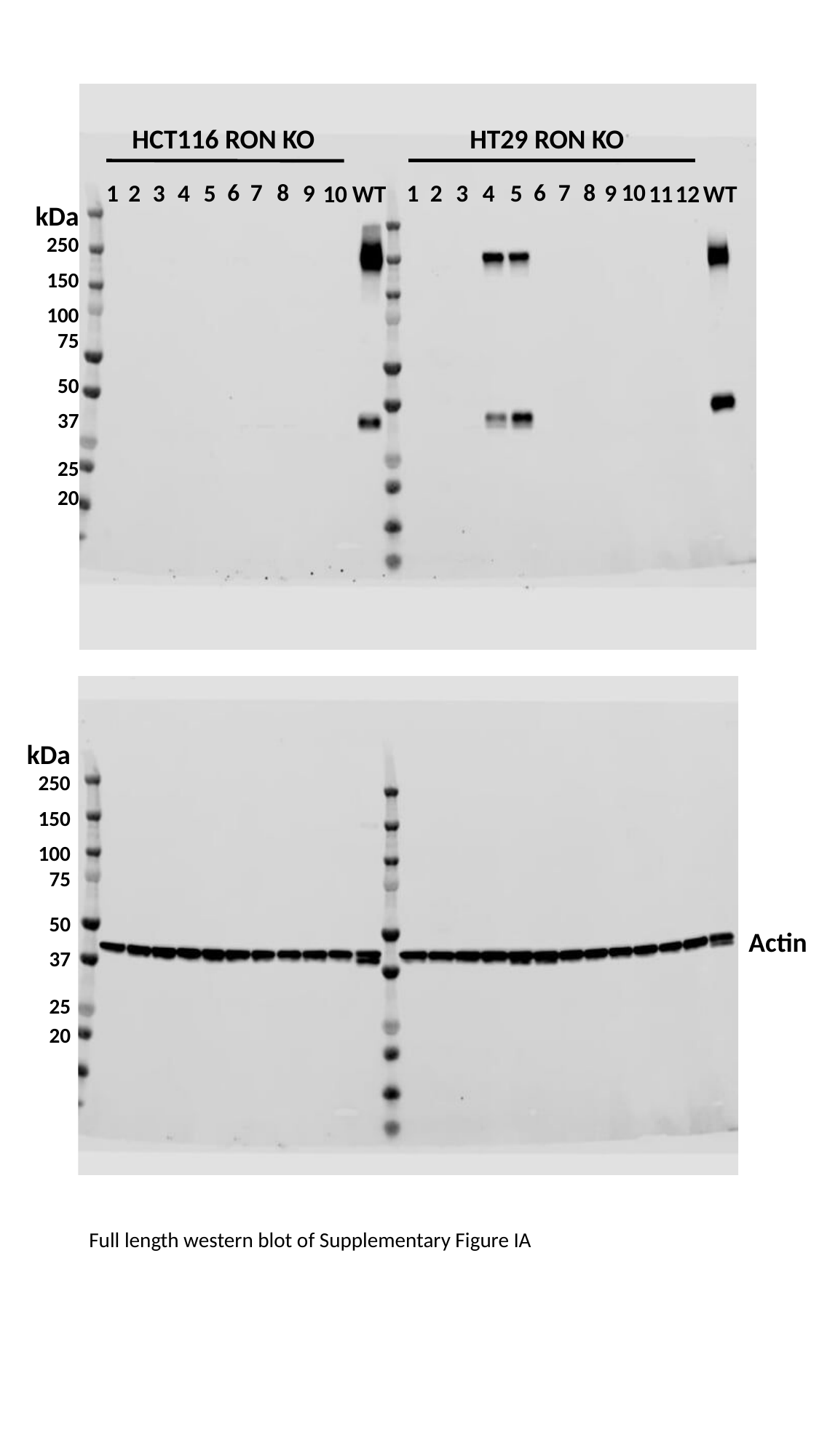

HCT116 RON KO
HT29 RON KO
10
6
7
8
6
7
8
9
9
5
5
2
3
4
2
3
4
1
1
10
WT
11
12
WT
kDa
250
150
100
75
50
37
25
20
kDa
250
150
100
75
50
Actin
37
25
20
Full length western blot of Supplementary Figure IA

## Slide 9
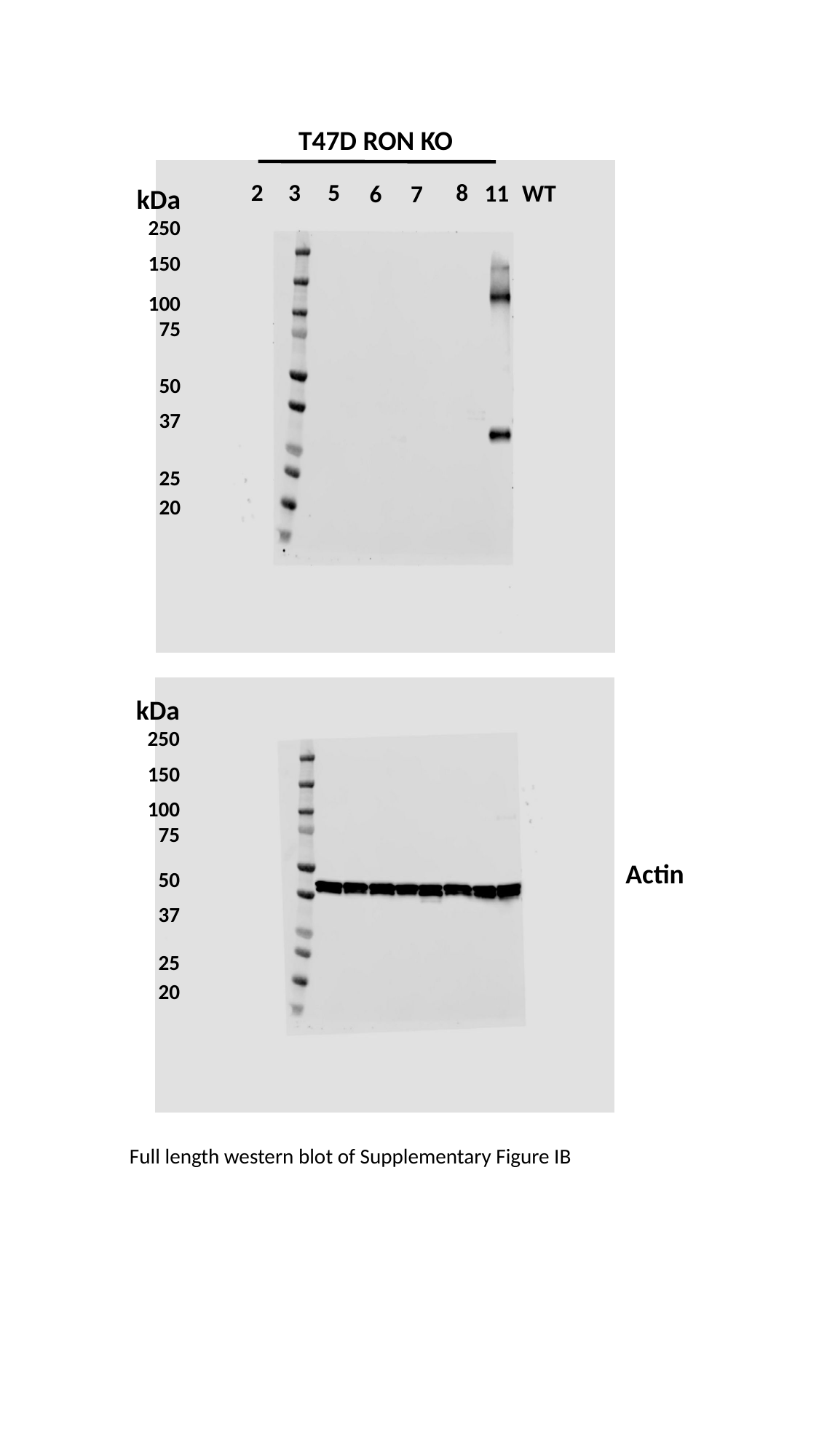

T47D RON KO
5
8
2
3
11
WT
6
7
kDa
250
150
100
75
50
37
25
20
kDa
250
150
100
75
Actin
50
37
25
20
Full length western blot of Supplementary Figure IB

## Slide 10
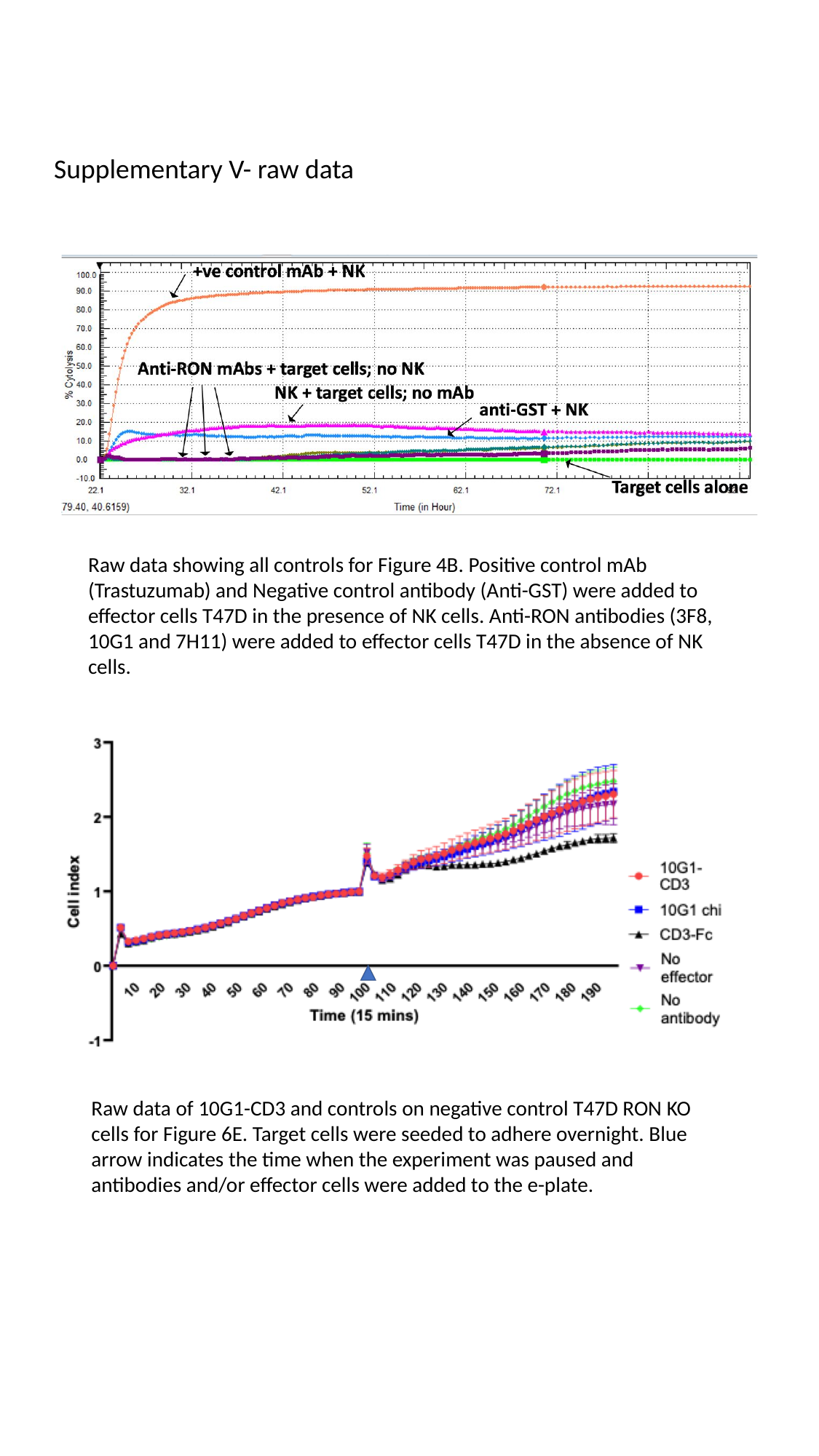

Supplementary V- raw data
Raw data showing all controls for Figure 4B. Positive control mAb (Trastuzumab) and Negative control antibody (Anti-GST) were added to effector cells T47D in the presence of NK cells. Anti-RON antibodies (3F8, 10G1 and 7H11) were added to effector cells T47D in the absence of NK cells.
Raw data of 10G1-CD3 and controls on negative control T47D RON KO cells for Figure 6E. Target cells were seeded to adhere overnight. Blue arrow indicates the time when the experiment was paused and antibodies and/or effector cells were added to the e-plate.
